# Annual and Analytical Cyclopædia of Practical Medicine

**Published:** 1900-01

**Authors:** 


ANNUAL AND ANALYTICAL CYCLOPÆDIA OF PRACTICAL MEDICINE. By Charles E. de M. Sajous, M.D., and One Hundred Associate Editors. Volume IV. Published by F. A. Davis Company, Philadelphia.

It is our pleasure to again call attention to this excellent work, the present being the fourth volume of Sajous' Annual. Like its three predecessors, it contains valuable information throughout its pages. This volume takes us from Diarrheal Diseases of Infants to Mercury, and while every subject shows an excellency throughout, some deserve especial mention. Among these are "Malarial Fevers," by Professor James C. Wilson and Dr. Thomas G. Ashton; "Locomotor Ataxia," by Dr. W. B. Pritchard of New York; "Intubation," by Professor W. E. Waxham of Chicago. We cannot but call attention to the value of this work as one of reference. Many physicians are located where they have no access to a medical library, but by possessing Sajous' Annual they obtain a small library in itself, with the advantage that it is thoroughly up to date. R.

